# Synthesis, structure and magnetocaloric properties of a new two-dimensional gadolinium(III) coordination polymer based on azo­benzene-2,2′,3,3′-tetra­carb­oxy­lic acid

**DOI:** 10.1107/S2053229621008871

**Published:** 2021-09-10

**Authors:** Wen-Wen Wei, Li-Ping Lu, Si-Si Feng, Miao-Li Zhu, Ulli Englert

**Affiliations:** a Institute of Molecular Science, Key Laboratory of Chemical Biology and Molecular Engineering of the Education Ministry, Shanxi University, 92 Wucheng Road, Taiyuan, Shanxi 030006, People’s Republic of China; b Institute of Inorganic Chemistry, RWTH Aachen University, Landoltweg. 1, Aachen 52074, Germany

**Keywords:** azo­benzenetetra­carb­oxy­lic acid, Gd^3+^ coordination polymer, bridging carboxyl­ate, anti­ferromagnetism, magnetocaloric effect, magnetic refrigeration, crystal structure

## Abstract

A new Gd^3+^ coordination polymer (CP), namely, poly[di­aqua­[μ_4_-1′-carb­oxy-3,3′-(diazene-1,2-di­yl)di­benzene-1,2,2′-tri­carboxyl­ato]gadolinium(III)], [Gd(C_16_H_7_N_2_O_8_)(H_2_O)_2_]_*n*_, (I), has been synthesized hydro­thermally from Gd(NO_3_)_3_·6H_2_O and azo­benzene-2,2′,3,3′-tetra­carb­oxy­lic acid (H_4_abtc). The target solid has been characterized by single-crystal and powder X-ray diffraction, elemental analysis, IR spectroscopy and susceptibility measurements. CP (I) crystallizes in the monoclinic space group *C*2/*c*. The structure features a 4-connected topology in which Gd^3+^ ions are connected by carboxyl­ate groups into a linear chain along the monoclinic symmetry direction. Adjacent one-dimensional aggregates are bridged by Habtc^3−^ ligands to form a two-dimensional CP in the (10) plane. A very short hydrogen bond [O⋯O = 2.4393 (4) Å] links neighbouring layers into a three-dimensional network. A magnetic study revealed anti­ferromagnetic Gd⋯Gd coupling within the chain direction. CP (I) displays a significant magnetocaloric effect (MCE), with a maximum −Δ*S*_m_ of 27.26 J kg^−1^ K^−1^ for Δ*H* = 7 T at 3.0 K. As the MCE in (I) exceeds that of the commercial magnetic refrigerant GGG (Gd_3_Ga_5_O_12_, −Δ*S*_m_ = 24 J kg^−1^ K^−1^, Δ*H* = 30 kG), CP (I) can be regarded as a potential cryogenic material for low-temperature magnetic refrigeration.

## Abstract 2

A novel coordination polymer (CP) has been successfully constructed under hydro­thermal conditions *via* the combination of Gd^3+^ ions and the azo­benzene-2,2′,3,3′-tetra­carb­oxy­lic acid linker. The underlying structural principles com­prise a 1D [Gd_2_(COO)_4_]_*n*_ chain and the linking of neighbouring chains *via* the organic ligand into a 2D structure with point symbol (4^4^·6^2^). Further crosslinking into a 3D framework occurs *via* very short hydrogen bonds. The new CP offers potential for application; magnetic studies reveal that it displays intra­chain anti­ferromagnetic Gd⋯Gd coupling and a cryogenic magnetocaloric effect.

## Introduction   

Coordination polymers (CPs), a class of com­pounds based on repetition of metal cations connected by coordinated linkers, have developed rapidly in the past 20 years (Chakraborty *et al.*, 2021 ▸) due to their inter­esting structures and variable applications in gas storage and separation (Roztocki *et al.*, 2020 ▸), catalysis (Kang *et al.*, 2019 ▸), sensing (Lustig *et al.*, 2017 ▸) and magnetic materials (Yang *et al.*, 2019*a* ▸). In particular, due to the unique 4*f* electron configuration of Ln^3+^ ions, lanthanide coordination polymers (Ln-CPs) usually exhibit a high coordination number, flexible coordination geometry and strong spin-orbit coupling (Sorace *et al.*, 2011 ▸; Liu *et al.*, 2016 ▸). These properties suggest their application in luminescence sensing (Ye *et al.*, 2017 ▸), mol­ecular magnetism (Liu *et al.*, 2019 ▸), magnetic resonance imaging (Debroye & Parac-Vogt, 2014 ▸) and related fields (Kumar *et al.*, 2019 ▸).

Magnetic refrigeration represents a focus area in the field of magnetism. This approach is based on the magnetocaloric effect (MCE) (Yang *et al.*, 2015 ▸; Wu *et al.*, 2021 ▸) and is considered a highly efficient and energy-saving, hence environmentally friendly, technology. Key factors for success com­prise a high-spin ground state *S*, negligible magnetic anisotropy and low-lying excited spin states (Evangelisti *et al.*, 2006 ▸; Liu *et al.*, 2014*a* ▸). The basic principle of magnetic refrigeration is realized through repeated cycles of isothermal magnetization and adiabatic demagnetization through the MCE displayed by the magnetic materials (Han *et al.*, 2018 ▸). Magnetic refrigeration has potential for the generation of ultra-low temperatures. The magnitude of the MCE is usually measured by magnetic entropy change (−Δ*S*_m_) and adiabatic temperature change (Δ*T*_ad_) under certain conditions (Franco *et al.*, 2018 ▸). A large Δ*S*_m_ under a relatively low magnetic field is mandatory for an attractive cryogenic magnetorefrigerant (Liu *et al.*, 2017 ▸). The −Δ*S*_m_ value of the well-known commercial low-temperature magnetic refrigeration material GGG (Gd_3_Ga_5_O_12_) is 24 J kg^−1^ K^−1^ (Δ*H* = 30 kG) (Daudin *et al.*, 1982 ▸).
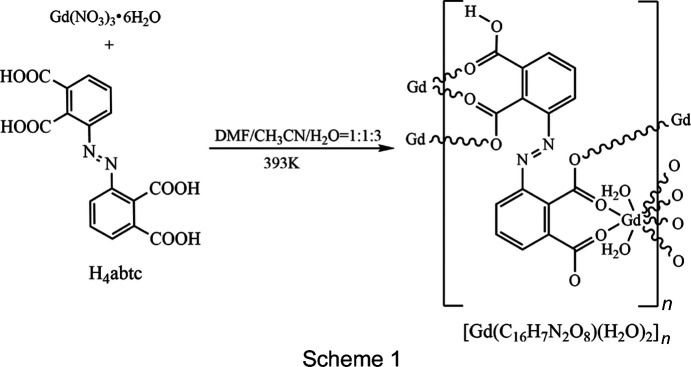


The Gd^3+^ ion meets the requirements of a high-spin ground state *S* (*S* = 7/2), of low-lying excited spin states and magnetic isotropy (Niu *et al.*, 2019 ▸). The magnetic coupling between Gd^3+^ centres is relatively weak, which allows the system to achieve a large MCE (Zhang *et al.*, 2021 ▸). Therefore, the Gd^3+^ ion represents an ideal choice for the construction of mol­ecular-based low-temperature magnetic refrigeration materials (Wang *et al.*, 2019 ▸). At present, mol­ecular materials of cryogenic magnetic refrigeration mainly include Gd-based clusters and Gd-based CPs. However, the exploration of MCE for one-dimensional (1D) linear Gd^3+^ CPs has only rarely been documented (Liu *et al.*, 2014*b* ▸).

In view of the above-mentioned promising properties, we report the new two-dimensional (2D) Gd^3+^ com­plex, [Gd(Habtc)(H_2_O)_2_]_*n*_, (I), for which we selected azo­benzene-2,2′,3,3′-tetra­carb­oxy­lic acid (H_4_abtc) as the ligand. The four carb­oxy­lic acid groups of this rigid H_4_abtc linker may be partially or com­pletely deprotonated and thus show flexible and diverse coordination patterns. In one of these coordination modes, the O atoms of a carboxyl­ate group can bridge Gd^3+^ ions and thus ensure magnetic exchange and transfer between adjacent Gd^3+^ ions, at the same time maintaining an overall rigid product (Zhang *et al.*, 2015*c* ▸). In this article, we communicate the synthesis, structure and magnetic properties of (I).

## Experimental   

All reagents and solvents used were commercially available and were used without further purification. H_4_abtc was purchased from Jinan Trading Company (China). FT–IR spectra were obtained with a Bruker TENSOR27 spec­trom­eter on KBr disks in the 4000–400 cm^−1^ region. Elemental analyses (EAs) were performed using a PerkinElmer 240 elemental analyzer. Powder X-ray diffraction (PXRD) data were collected on a Bruker D8 Advance X-ray diffractometer (Cu *K*α, λ = 1.5418 Å) at a rate of 10° min^−1^ in the 2θ range 5–50°. Based on the results of the single-crystal X-ray dif­frac­tion experiment, the simulated pattern was obtained with *Mercury* (Macrae *et al.*, 2020 ▸) assuming Cu *K*α_1_ radiation (λ = 1.54056 Å). The thermogravimetric analysis was performed on a Dupont thermal analyzer between room temperature and 1045 K under an N_2_ flow with a heating rate of 10 K min^−1^. Magnetic susceptibility was measured from a microcrystalline sample using a SQUID magnetometer (Quantum Design MPMS) in the range 2–300 K with a direct-current field of 1000 Oe. Isothermal field-dependent magnetization *M*(*H*) was measured in the range 0–7 T from 2 to 10 K.

### Synthesis and crystallization   

The reaction route to (I) is shown in Scheme 1. Gd(NO_3_)_3_·6H_2_O (67.7 mg, 0.15 mmol) and H_4_abtc (35.8 mg, 0.1 mmol) were dissolved in a mixture of *N*,*N*-di­methyl­formamide (DMF, 2 ml), aceto­nitrile (CH_3_CN, 2 ml) and dis­tilled water (H_2_O, 6 ml). The solution was sealed in a stainless steel container and heated under autogenous pressure at 393 K for 72 h. After this period, heating was sus­pended and the container was allowed to cool to room tem­per­ature. Yellow block-shaped crystals of the product were obtained by filtration, washed with water and dried in the air (yield 67%). Analysis calculated (%) for C_16_H_11_GdN_2_O_10_: C 35.01, H 2.01, N 5.10; found: C 35.05, H 2.02, N 5.13.

### Refinement   

**Figure 1:**
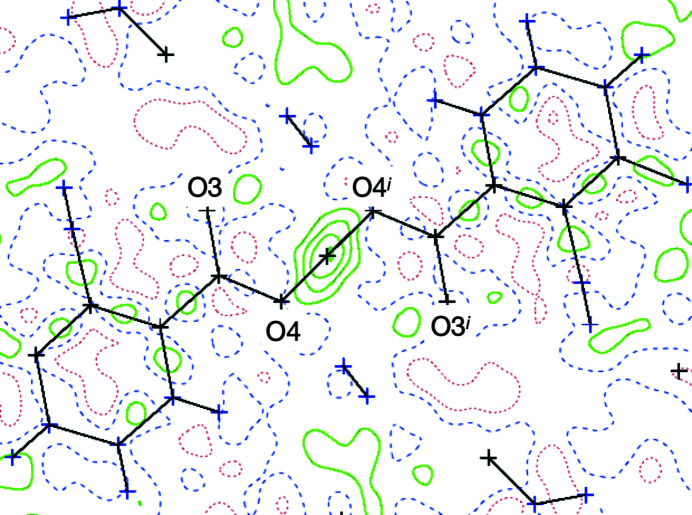
Difference Fourier map (*PLATON*; Spek, 2020 ▸) for (I) before inclusion of H4*A* into the structure model. Contour lines are drawn at an electron density of 0.1 e Å^−3^. [Symmetry code: (i) −*x*, −*y* + 1, −*z* + 1.]

Crystal data, data collection and structure refinement details are summarized in Table 1 ▸. Carbon-bound H atoms were placed in calculated positions and refined using a riding model, with aromatic C—H = 0.93 Å and *U*_iso_(H) = 1.2*U*_eq_(C). The water H-atom positions were fixed as found (O—H dis­tances are approximately 0.82 Å), with *U*_iso_(H) = 1.5*U*_eq_(O). A difference Fourier map (Fig. 1 ▸) suggested Wyckoff position 4*b* for atom H4*A* in the short O⋯O contact, albeit as a very broad residual electron-density maximum. Our structure model with H4 in this special position therefore assumes a short symmetric hydrogen bond. In the absence of high-resolution or neutron data, we can neither disprove nor support a split-atom alternative and an asymmetric hydrogen bond. Şerb *et al.* (2011 ▸) have com­piled structures featuring very short O⋯O bonds. The reflection conditions for the correct space group *C*2/*c* are also com­patible with the subgroup *Cc*; tentative refinements in this noncentrosymmetric subgroup resulted in numerous high correlations and anti­correlations for positional and displacement parameters: 26 elements of the final inverted refinement matrix showed correlation coefficients with a modulus >0.9 and more than 100 with a modulus >0.8. These high correlations resulted in an unrealistically broad range of C—C bonds, and no convergence for physically meaningful displacement parameters could be achieved.

## Results and discussion   

### IR spectroscopy   

**Figure 2:**
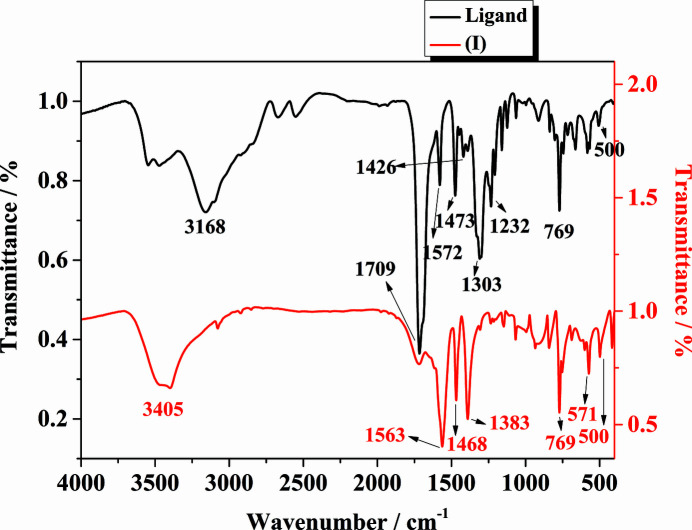
IR spectra of the ligand and (I).

The IR spectra of the ligand and (I) in the range 4000–400 cm^−1^ are presented in Fig. 2 ▸. The broad band at 3405 cm^−1^ indicates O—H stretching of the hy­droxy groups and the coordinated water mol­ecules in (I) (Yang *et al.*, 2019*b* ▸). The characteristic absorption peaks of the asymmetric and symmetric stretching vibrations of the carboxyl­ate groups appear at 1383 and 1563 cm^−1^ for (I) (Du *et al.*, 2016 ▸; Li *et al.*, 2012 ▸; Zhang *et al.*, 2015*a* ▸). They are clearly shifted to lower wavenumbers in com­parison with free H_4_abtc (1426 and 1572 cm^−1^), suggesting that the carboxyl­ate groups in the com­plex are coordinated to the Gd^3+^ ions (An *et al.*, 2018 ▸). The absorption observed at 1468 cm^−1^ is caused by the N=N stretching vibration of the ligand (Goel & Kumar, 2018 ▸). The structural features of the com­plex deduced from IR spectra match the results of the single-crystal X-ray analysis. IR (KBr, ν, cm^−1^, *s* = strong, *m* = medium and *w* = weak): 3405 (*m*), 1709 (*w*), 1563 (*s*), 1468 (*s*), 1383 (*s*), 1298 (*w*), 1147 (*w*), 1072 (*m*), 934 (*w*), 840 (*m*), 769 (*s*), 684 (*w*), 571 (*s*), 500 (*s*).

### Structure description   

**Figure 3:**
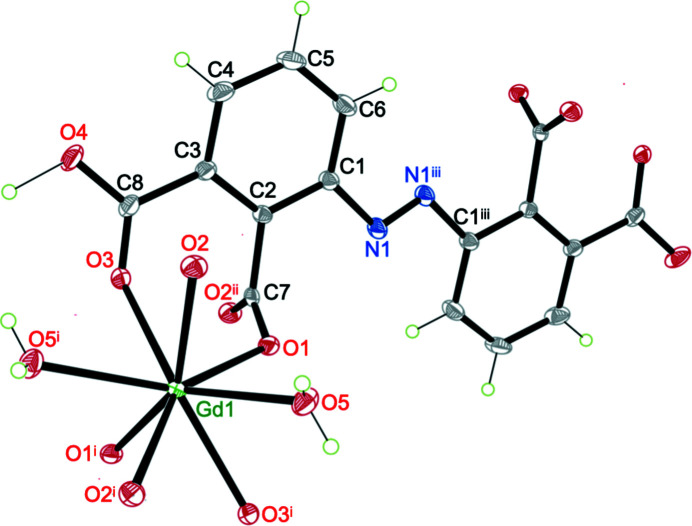
Expanded asymmetric unit and coordination environment of the Gd^3+^ ion in (I). Displacement ellipsoids are drawn at 30% probability and H atoms are represented as spheres of arbitrary radius. [Symmetry codes: (i) −*x*, *y*, −*z* + 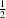; (ii) *x*, *y* + 1, *z*; (iii) −*x* + 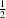. −*y* + 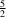, −*z* + 1.]

**Figure 4:**
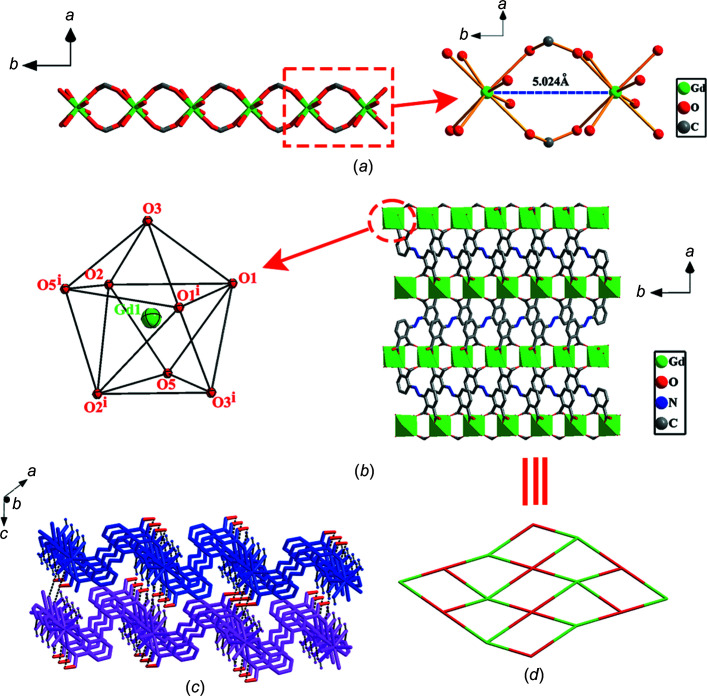
(*a*) Distances between adjacent Gd^3+^ ions in the 1D metal chain constructed by Gd^3+^ ions and the carboxyl­ate groups of the Habtc^3−^ ligands (H atoms have been omitted for clarity). (*b*) The 2D layer of (I). The inset is the local coordination geometry of the Gd^3+^ ion of (I). [Symmetry code: (i) −*x*, *y*, −*z* + 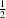.] (*c*) The 3D framework formed by hydrogen bonds in (I) (different colours represent different layers and H4*A* atoms are shown in red). (*d*) The 2D topology of (I) with point symbol (4^4^·6^2^).

Coordination polymer (I) crystallizes in the monoclinic space group *C*2/*c*, adopting a 2D framework based on coordination and covalent bonds; we originally expected a three-dimensional (3D) solid from the reaction between Gd(NO_3_)_3_·6H_2_O and H_4_abtc. The asymmetric unit of (I) contains a Gd^3+^ ion situated on a twofold axis (Wyckoff position 4*e*), one half of the Habtc^3−^ ligand and a coordinated H_2_O mol­ecule. As shown in Fig. 3 ▸, each Gd^3+^ ion is eight-coordinated by O atoms in a {GdO_8_} environment, in which six O atoms [O1, O2, O3, O1^i^, O2^i^ and O3^i^; symmetry code: (i) −*x*, −*y* + 1, −*z* + 1] are derived from the carboxyl­ate groups of four Habtc^3−^ moieties and two O atoms (O5 and O5^i^) represent aqua ligands. The Gd—O distances are in the range 2.3449 (15)–2.4503 (16) Å and the O—Gd—O angles vary from 68.66 (5) to 149.37 (5)° (Table 2 ▸), consistent with values observed in related com­pounds (Nakamura *et al.*, 2021 ▸). The coordination polyhedron about the Gd^3+^ ion displays a dicapped trigonal prismatic geometry, in which each Habtc^3−^ links four Gd^3+^ ions in a μ_4_-η^1^:η^1^:η^1^:η^0^:η^1^:η^1^:η^1^:η^0^ coordination mode and all Gd^3+^ ions are connected *via* four bridging Habtc^3−^ ligands. Adjacent Gd^3+^ atoms are linked by the carboxyl­ate groups of Habtc^3−^, forming a linear Gd chain along [010]; the Gd⋯Gd separation corresponds to the lattice parameter *b* of 5.0236 (9) Å [Fig. 4 ▸(*a*)]. The 1D Gd chains are bridged by the central azo group of the Habtc^3−^ ligands to form a layer structure [Fig. 4 ▸(*b*)]. Two Habtc^3−^ ligands share the proton H4 which is located on a centre of inversion [see *Refinement* (§2.2) and Fig. 1 ▸] and plays the decisive role in linking adjacent coordination layers to a 3D framework [Fig. 4 ▸(*c*)]. In addition to this very short and symmetric hydrogen bond, the aqua ligand O5 acts as a hydrogen-bond donor towards carboxyl­ate O atoms of a neighbouring layer. Detailed information of the inter­molecular hydrogen bonds is summarized in Table 3 ▸. In order to obtain better insight into the nature of the intricate structure of CP (I), the network was simplified and its topology was analyzed with the help of the program *TOPOS* (Blatov & Shevchenko, 2006 ▸). As shown in Fig. 4 ▸(*d*), each Habtc^3−^ ligand can be perceived a four-connected node towards Gd^3+^ ions and, *vice versa*, each Gd^3+^ ion is coordinated by four Habtc^3−^ ligands. The overall network can thus be described as a 4-connected net with the point symbol (4^4^·6^2^).

### Powder X-ray diffraction (PXRD) and thermal stability   

**Figure 5:**
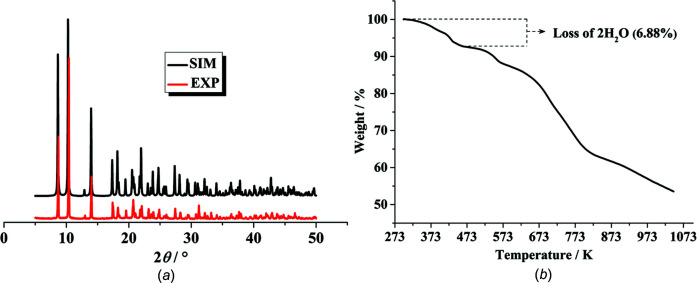
(*a*) Experimental and simulated PXRD patterns of (I) in the range 5–50°. (*b*) Thermogravimetric analysis for (I).

To verify the phase purity of the com­pound, the as-syn­the­sized samples were characterized by PXRD at room temperature. As shown in Fig. 5 ▸(*a*), the experimental PXRD pattern of (I) is in excellent agreement with the simulated one, demonstrating the phase purity of the bulk sample. Minor differences in line intensities can probably be attributed to preferred orientation of the powder sample. Thermal stability was investigated by a thermogravimetric analysis (TGA) under an N_2_ atmosphere. Fig. 5 ▸(*b*) summarizes the weight loss for (I) between room temperature and 1045 K. In the temperature range 325–471 K, the TGA curve shows a weight loss of 6.88% which may be attributed to the elimination of two coordinated water mol­ecules (calculated 6.56%). At higher temperatures, the framework of (I) gradually collapses.

### Magnetic properties   

**Figure 6:**
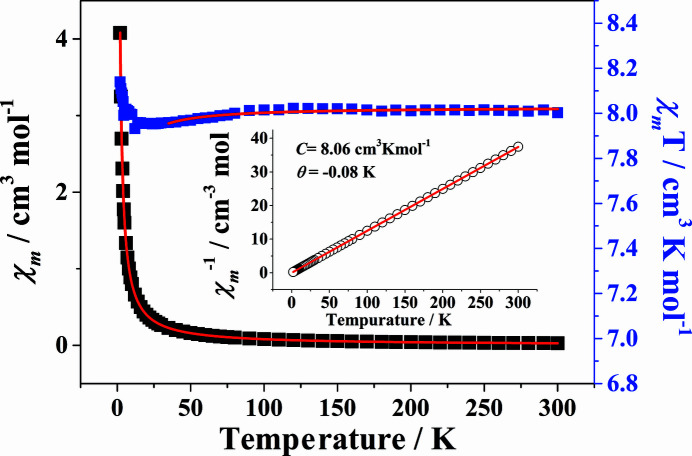
Plots of χ_m_, χ_m_ *T* and χ_m_^−1^ (inset) as functions of *T* for (I). Red solid lines represent best fits.

Magnetic properties of (I) were studied in order to understand potential magnetic inter­actions. Variable-temperature magnetic susceptibility measurements of (I) were conducted in the range 2–300 K with an applied magnetic field of 1000 Oe. As shown in Fig. 6 ▸, the experimental χ_m_ *T* value for (I) amounts to 8.00 cm^3^ mol^−1^ K at 300 K, close to the expected value of 7.88 cm^3^ mol^−1^ K calculated for an isolated Gd^3+^ ion (*S* = 7/2, *g* = 2) (Xi *et al.*, 2020 ▸). As the temperature is decreased, the χ_m_ *T* value of (I) decreases slowly to 7.93 cm^3^ mol^−1^ K around 10 K, and then increases gradually to 8.14 cm^3^ mol^−1^ K at 2 K. The data in the whole temperature range 2–300 K fit well the Curie–Weiss law with *C* = 8.06 cm^3^ K mol^−1^ and θ = −0.08 K. The negative θ value indicates the existence of weak anti­ferromagnetic inter­actions between the metal centres in the 1D chain of (I). To further qu­anti­tatively analyze the magnetic inter­actions, the molar susceptibility of (I) can be described by a Fisher expression for a classical spin chain which allows an evaluation of the mag­netic coupling (*J*) between adjacent Gd^3+^ ions (Farger *et al.*, 2018 ▸). The best least-squares fit parameters are *g* = 2.01 and *J* = −0.02 cm^−1^, with an agreement factor *R* = 6.27 × 10^−5^ in the range 35–300 K. The value for *J* further proves the existence of weak anti­ferromagnetic inter­actions between adjacent Gd^3+^ ions in (I).

**Figure 7:**
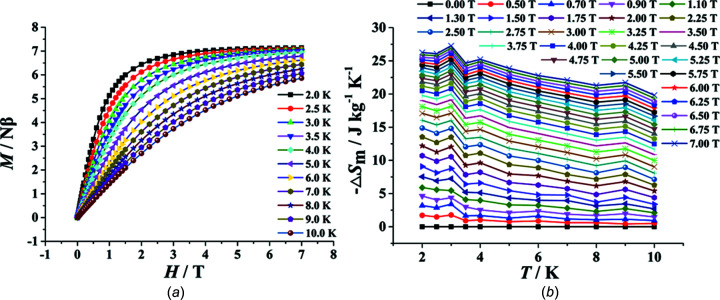
(*a*) *M versus H* plots from 2 to 10 K. (*b*) Calculated −Δ*S*_m_ from the magnetization data of (I) at various fields and temperatures.

The magnetization of (I) was measured in the inter­val between 0 and 7 T at temperatures between 2 and 10 K (Fig. 7 ▸ *a*). The *M* values for (I) show a steady increase with increasing *H* and a saturation value of 7.14 Nβ at 7 T and 2 K, which is close to the expected value of *S*×*g* = 7/2×2 = 7 Nβ for an isolated Gd^3+^ ion (*S* = 7/2, *g* = 2). To evaluate the magnetocaloric effect (MCE), the magnetic entropy change (−Δ*S*_m_) of (I) was calculated for a field between 0 and 7 T in the temperature range 2–10 K, and it can be obtained (Fig. 7 ▸ *b*) by the Maxwell relation in the equation Δ*S*_m_(*T*) = [*M*(*T*,*H*)/*T*]_*H*_d*H*. The resulting maximum value of −Δ*S*_m_ amounts to 27.26 J kg^−1^ K^−1^ for Δ*H* = 7 T at 3.0 K, which is smaller than the theoretical value of 31.52 J kg^−1^ K^−1^, as calculated from the equation −Δ*S*_m_ = *N*_Gd_ *R*ln(2*s* + 1)/*M*_W_, with *S* = 7/2. In this equation, *M*_W_ is the formula mass of 548.52 g mol^−1^ and *N*_Gd_ is the number of Gd^3+^ ions present per mole of (I). The difference in −Δ*S*_m_ between the theoretical and experimental values may be attributed to the existence of anti­ferromagnetic inter­actions between Gd^3+^ ions. The experimental −Δ*S*_m_ value is also smaller than several previously prepared 1D linear-chain Gd^3+^ com­plexes (Table 4 ▸), which can be ascribed to the large *M*_W_/*N*_Gd_ ratio arising from the large H_4_abtc ligand and the anti­ferromagnetic inter­actions between the neighbouring Gd^3+^ ions in (I).

### Conclusion   

In summary, the novel coordination polymer (I) has been successfully constructed under hydro­thermal conditions *via* the combination of Gd^3+^ ions and the H_4_abtc linker. The underlying structural principles in (I) com­prise a 1D [Gd_2_(COO)_4_]_*n*_ chain and the linking of neighbouring chains *via* the organic ligand into a 2D structure with point symbol (4^4^·6^2^). Further crosslinking into a 3D framework occurs *via* very short hydrogen bonds. The new CP offers potential for application; magnetic studies reveal that (I) displays intra­chain anti­ferromagnetic Gd⋯Gd coupling and a cryogenic MCE with the maximum −Δ*S*_m_ of 27.26 J kg^−1^ K^−1^ for Δ*H* = 7 T at 3.0 K. This small −Δ*S*_m_ value can be ascribed to the high *M*_W_/*N*_Gd_ ratio arising from the large H_4_abtc ligand and the anti­ferromagnetic inter­actions between neighbouring Gd^3+^ ions in (I). The selection of low mol­ecular-weight ligands that transfer weak coupling may be a promising approach for obtaining Gd^3+^ com­plexes as mol­ecule-based magnetic refrigerants. Further studies on Gd^3+^ com­plexes for magnetic refrigeration are underway in our laboratory.

## Supplementary Material

Crystal structure: contains datablock(s) I, global. DOI: 10.1107/S2053229621008871/cu3174sup1.cif

Structure factors: contains datablock(s) I. DOI: 10.1107/S2053229621008871/cu3174Isup2.hkl

CCDC reference: 2105211

## Figures and Tables

**Table 1:** Experimental details

Crystal data	
Chemical formula	[Gd(C_16_H_7_N_2_O_8_)(H_2_O)_2_]
*M*_r_	548.52
Crystal system, space group	Monoclinic, *C*2/*c*
Temperature (K)	298
*a*, *b*, *c* (Å)	25.725 (4), 5.0236 (9), 17.274 (3)
β (°)	127.393 (4)
*V* (Å^3^)	1773.6 (5)
*Z*	4
Radiation type	Mo *K*α
μ (mm^−1^)	3.80
Crystal size (mm)	0.20 × 0.15 × 0.15

Data collection	
Diffractometer	Bruker APEXII CCD
Absorption correction	Multi-scan (*SADABS*; Krause *et al.*, 2015 ▸)
*T*_min_, *T*_max_	0.600, 0.747
No. of measured, independent and observed [*I* > 2σ(*I*)] reflections	6278, 1568, 1508
*R*_int_	0.024
(sin θ/λ)_max_ (Å^−1^)	0.598

Refinement	
*R*[*F*^2^ > 2σ(*F* ^2^)], *wR*(*F* ^2^), *S*	0.013, 0.034, 1.11
No. of reflections	1568
No. of parameters	133
H-atom treatment	H atoms treated by a mixture of independent and constrained refinement
Δρ_max_, Δρ_min_ (e Å^−3^)	0.29, −0.33

Computer programs: *APEX2* (Bruker, 2009 ▸), *SAINT* (Bruker, 2009 ▸), *SHELXS97* (Sheldrick, 2008 ▸), *SHELXL2014* (Sheldrick, 2015 ▸) and *SHELXTL* (Bruker, 2009 ▸).

**Table 2:** Selected geometric parameters (Å, °)

Gd1—O1	2.3449 (15)	Gd1—O3	2.4446 (15)
Gd1—O2	2.3722 (15)	Gd1—O5	2.4502 (16)
			
O1—Gd1—O1^i^	88.98 (8)	O3—Gd1—O3^i^	139.00 (8)
O1—Gd1—O2	92.22 (6)	O1—Gd1—O5^i^	140.25 (5)
O1—Gd1—O2^i^	149.36 (5)	O1—Gd1—O5	80.69 (6)
O2—Gd1—O2^i^	101.87 (8)	O2—Gd1—O5^i^	78.93 (6)
O1—Gd1—O3	71.73 (5)	O2—Gd1—O5	69.85 (6)
O1—Gd1—O3^i^	79.27 (5)	O3—Gd1—O5^i^	68.66 (5)
O2—Gd1—O3	72.12 (5)	O3—Gd1—O5	131.45 (5)
O2—Gd1—O3^i^	138.46 (5)	O5—Gd1—O5^i^	129.63 (9)

Symmetry code: (i) -x, y, -z+{\script{1\over 2}}.

**Table 3:** Hydrogen-bond geometry (Å, °)

*D*—H⋯*A*	*D*—H	H⋯*A*	*D*⋯*A*	*D*—H⋯*A*
O4—H4*A*⋯O3^ii^	1.22	2.42	3.130 (2)	114
O4—H4*A*⋯O4^ii^	1.22	1.22	2.439 (4)	180
O5—H5*A*⋯O1^iii^	0.82	2.03	2.756 (2)	147
O5—H5*B*⋯O4^iv^	0.82	2.01	2.821 (3)	173

Symmetry codes: (ii) -x, -y+1, -z+1; (iii) x, y-1, z; (iv) x, -y+1, z-{\script{1\over 2}}.

**Table 4:** Comparison of −Δ*S*_m_ for (I) and several previously reported 1D Gd^3+^ com­plexes OAc is acetate, pda is propanedionate, ox is oxalate, cit is citrate, piv is pivalate, MMA is methylmalonate, INA is isonicotinate, glu is glutamate, HPA is homophtalate, azdc is 4,4′-azobenzoate, phen is 1,10-phenanthroline, 2,5-TDA is thiophene-2,5-dicarboxylate, DMA is dimethylacetamide, DMF is dimethylformamide, N-BDC is 2-aminobenzene-1,4-dicarboxylate, mnba is *m*-nitrobenzoate, PAA is phenylacetate, HIN is isonicotinic acid and IN is isonicotinate.

Complex	Dimensionality	−Δ*S*_m_^max^ ( J kg^−1^ K^−1^)	Gd⋯Gd (Å)	*M*_W_/*N*_Gd_	Reference
[Gd(OAc)_3_(H_2_O)_0.5_]_*n*_	One-dimensional	50.4	4.0	343	Guo *et al.* (2012 ▸)
[Gd(pda)(ox)_0.5_]_*n*_	Three-dimensional	46.8	4.1–6.1	303	Liu *et al.* (2017 ▸)
[Gd(pda)(ox)_0.5_(H_2_O)]_*n*_	Three-dimensional	46.1	4.3–6.3	321	Liu *et al.* (2017 ▸)
[Gd(HCOO)(OAc)_2_(H_2_O)_2_]_*n*_	One-dimensional	45.9	5.9	572	Lorusso *et al.* (2012 ▸)
[Gd(OAc)_3_(MeOH)]_*n*_	One-dimensional	45.0	4.1	366	Guo *et al.* (2012 ▸)
[Gd(pda)(ox)_0.5_(H_2_O)_2_]_*n*_	Two-dimensional	45.0	4.2–6.2	339	Liu *et al.* (2017 ▸)
[Gd(cit)(H_2_O)]_*n*_	Two-dimensional	43.6	4.5	363	Liu *et al.* (2014*b* ▸)
[Gd_2_(piv)_5_(μ_3_-OH)(H_2_O)]_*n*_	One-dimensional	37.5	3.7	427	Liu *et al.* (2014*b* ▸)
[Gd(MMA)(INA)(H_2_O)_2_]_*n*_	Two-dimensional	36.0	4.7	431	Li *et al.* (2017*a* ▸)
{[Gd_2_(glu)_3_(H_2_O)_2_]·4H_2_O}_*n*_	Three-dimensional	36.0	4.2	406	Zheng *et al.* (2017 ▸)
{[Gd(HPA)(NO_3_)(H_2_O)_2_]·H_2_O}_*n*_	One-dimensional	35.6	3.9	415	Li *et al.* (2017*b* ▸)
{[Gd_2_(HPA)_3_(H_2_O)_2_]·H_2_O}_*n*_	Two-dimensional	35.4	3.9	415	Li *et al.* (2017*b* ▸)
[Gd(azdc)(HCOO)]_*n*_	Three-dimensional	34.9	3.9	470	Zhang *et al.* (2015*b* ▸)
[Gd_2_(MMA)_2_(INA)_2_(H_2_O)_3_]_*n*_	Two-dimensional	34.3	4.8	844	Li *et al.* (2017*a* ▸)
[Gd_2_(SO_4_)_3_(phen)_2_(H_2_O)_2_]_*n*_	One-dimensional	31.7	4.3	499	Zheng *et al.* (2017 ▸)
[Gd_2_(2,5-TDA)_3_(DMA)_2_]_*n*_	Three-dimensional	31.0	4.1	499	Kumar *et al.* (2020 ▸)
{[Gd_2_(OH)_2_ *L*_2_]·DMF·4H_2_O}_*n*_	Three-dimensional	30.3	3.8–3.9	417	Peng *et al.* (2018 ▸)
[Gd_2_(N-BDC)_3_(DMF)_4_]_*n*_	Three-dimensional	29.0	10.5–12.1	366	Lorusso *et al.* (2012 ▸)
[Gd*L*_1/2_(H_2_O)_2_]_*n*_	Two-dimensional	27.3	5.0	548	This work
[Gd_2_(mnba)_4_(μ-OH)_2_(H_2_O)]_*n*_	One-dimensional	27.1	3.8	515	Liu *et al.* (2014*b* ▸)
[Gd(PAA)_3_(H_2_O)]_*n*_	One-dimensional	26.7	4.0	580	Li *et al.* (2017*c* ▸)
{Gd[IN][HIN][CH_2_OCH_2_O]}_*n*_	One-dimensional	26.2	3.7	462	Li *et al.* (2020 ▸)
{[Gd_2_(azdc)_3_(DMA)_2_]·2DMA}_*n*_	Three-dimensional	22.3	4.6	734	Zhang *et al.* (2014 ▸)
